# New Reference Genome Sequences for 17 Bacterial Strains of the Honey Bee Gut Microbiota

**DOI:** 10.1128/MRA.00834-18

**Published:** 2018-07-26

**Authors:** Kirsten M. Ellegaard, Philipp Engel

**Affiliations:** a Department of Fundamental Microbiology, University of Lausanne, Lausanne, Switzerland; Louisiana State University

## Abstract

We sequenced the genomes of 17 strains isolated from the gut of honey bees, including strains representing the genera Lactobacillus, Bifidobacterium, Gilliamella, Snodgrassella, Frischella, and Commensalibacter. These genome sequences represent an important step forward in the development of a comprehensive reference database to aid future analysis of this emerging gut microbiota model.

## ANNOUNCEMENT

The honey bee gut is colonized by a remarkably simple community dominated by only 8 to 10 bee-specific phylotypes (1). However, genome-level analyses have shown that several of the phylotypes comprise highly divergent strains (2–4). As such, the honey bee is a promising future model for studying strain-level evolution and function in gut-associated bacterial communities (5). Here, we present 17 new genome sequences of strains isolated from the gut of honey bees, which were generated to facilitate the development of a reference genome database for this community. All strains were isolated from honey bees collected from our apiary in Lausanne, Switzerland, by culturing gut homogenates on agar plates (6) under microaerophilic or anaerobic conditions (7).

Four strains of the genus Lactobacillus (Table 1) were selected for sequencing with PacBio 20K (Pacific Biosciences) single-molecule real-time (SMRT) technology. The strains were grown overnight in MRS broth supplemented with fructose and cysteine (8) at 35°C under anaerobic conditions, and total genomic DNA was extracted using a cetyltrimethylammonium bromide-based extraction protocol (7). *De novo* genome assembly was done using the Hierarchical Genome Assembly Process (HGAP) version 2.3. Another 13 strains representing the genera Bifidobacterium, Gilliamella, Snodgrassella, Frischella, and Commensalibacter (Table 1) were selected for sequencing with Illumina technology. The strains were cultured as described previously (7), and total genomic DNA was extracted with the GenElute bacterial genomic DNA kit according to the manufacturer’s instructions (Table 1). Sequencing libraries were prepared with the TruSeq DNA kit and sequenced on the MiSeq platform (Illumina) using the paired-end 2 × 250-bp protocol. All 13 genomes were sequenced to a minimum depth of 50× (Table 1). The resulting FASTQ files were trimmed with Trimmomatic (9) to remove eventual adapter sequences and low-quality reads using the following parameters: LEADING, 20; TRAILING, 20; SLIDINGWINDOW, 4:15; and MINLEN, 50. The reads were assembled with SPAdes version 3.7.1 (10) using the “-careful” flag and multiple *k*-mer sizes (21, 33, 55, 77, 99, 127). Small contigs (less than 500 bp) and contigs with low *k*-mer coverage (less than 5×) were removed from the assemblies, resulting in 6 to 40 contigs per assembly, with a median *N*_50_ of 529,190 bp. For strains with related complete genome sequences or scaffolds available, the contigs were reordered with Mauve (11).

**TABLE 1:** Genome assembly statistics and strain information

Genus	Species	Phylotype^*a*^	Sublineage	Strain	Extracted DNA (µg)^*b*^	No. of contigs	*N*_50_ (bp)	Assembly size (bp)	Coverage (×)	GC content (%)	No. of genes^*c*^
Lactobacillus	L. apis	Firm5	Firm5-1	ESL0185	17.9	1	1,683,102	1,683,102	420	37	1,578
Lactobacillus	L. helsingborgensis	Firm5	Firm5-2	ESL0183	19.3	2	1,856,015	1,867,232	300	37	1,780
Lactobacillus	L. melliventris	Firm5	Firm5-3	ESL0184	19.8	4	1,505,590	2,036,181	320	36	2,015
Lactobacillus	L. kulllabergensis	Firm5	Firm5-4	ESL0186	16.1	1	2,018,944	2,018,944	290	36	1,915
Bifidobacterium	B. asteroides	Bifido	Bifido-1	ESL0170	1.1	7	1,162,986	2,175,262	200	60	1,771
Bifidobacterium	B. asteroides	Bifido	Bifido-1	ESL0198	1.3	12	618,428	2,235,610	280	60	1,820
Bifidobacterium	B. asteroides	Bifido	Bifido-1	ESL0199	5.3	7	558,059	2,167,340	50	59	1,741
Bifidobacterium	B. asteroides	Bifido	Bifido-1	ESL0200	4.7	16	500,320	1,933,421	300	60	1,621
Bifidobacterium	B. indicum / B. coryneforme	Bifido	Bifido-2	ESL0197	1.1	6	1,389,647	1,715,238	300	61	1,408
Gillamella	G. apicola	Gilliamella	Gilli-1	ESL0178	0.3	18	364,598	2,885,657	200	34	2,602
Gillamella	G. apis	Gilliamella	Gilli-2	ESL0169	3.9	13	481,163	2,430,778	270	35	2,227
Gillamella	G. apis	Gilliamella	Gilli-2	ESL0172	1.8	17	374,672	2,685,772	200	34	2,468
Gillamella	NA^*d*^	Gilliamella	Gilli-3	ESL0177	2.0	19	953,736	3,086,198	50	35	2,868
Gillamella	NA	Gilliamella	Gilli-3	ESL0182	1.2	31	255,373	3,537,173	160	35	3,257
Snodgrassella	S. alvi	Snodgrasella	NA	ESL0196	3.7	15	1,281,809	2,446,304	130	41	2,224
Frischella	F. perrara	Frischella	NA	ESL0167	3.1	40	277,847	2,558,525	200	34	2,313
Commensali- bacter	Commensalibacter sp.	Commensalibacter	NA	ESL0284	1.3	13	471,180	1,948,862	50	38	1,767

a Based on 16S rRNA amplicon sequencing.

b Total amount of extracted DNA.

c Gene count based on the JGI Microbial Genome Annotation Pipeline.

d NA, not applicable.

Assembly qualities were checked by remapping reads to assemblies with the Burrows-Wheeler Aligner (12) and by GC-skew visualization with DNAplotter (13). For strain ESL0184, the main chromosome was cut into three contigs due to assembly uncertainty generated by a duplicated prophage sequence. Strains ESL0183, ESL0185, and ESL0186 were submitted as complete genomes, with strain ESL0183 having a small plasmid contig of 11.3 kb.

Core phylogenies were generated for the Lactobacillus, Bifidobacterium, and Gilliamella strains, including previously published isolates derived from honey bees, using OrthoFinder (14) for ortholog prediction and RAxML (15) for phylogenetic inference. Based on the phylogenies, the Lactobacillus and Bifidobacterium strains represent members of previously reported sublineages, whereas two strains of the genus Gilliamella (ESL0177 and ESL0182) represent a new sublineage, with strain ESL182 having the largest genome size reported for this genus to date (3.5 Mbp) (Table 1).

### Data availability.

The complete genome sequences for the strains reported here have been deposited in GenBank under the accession numbers CP029476, CP029544/CP029545, and CP029477, and the whole-genome shotgun projects have been deposited under the accession numbers QGLH00000000, QGLJ00000000, QGLK00000000, QGLL00000000, QGLI00000000, QGLG00000000, QGLQ00000000, QGLN00000000, QGLO00000000, QGLP00000000, QGLR00000000, QGLS00000000, QGLM00000000, and QGLT00000000. Additionally, the genomes were annotated using the JGI Microbial Genome Annotation Pipeline, where they have been deposited under the genome identification numbers 2684622912, 2684622914, 2684622911, 2684622916, 2684622918, 2684622919, 2684622920, 2684622917, 2684622913, 2684622925, 2684622922, 2684622923, 2684622924, 2684622926, 2684622927, 2684622921, and 2756170209.
